# The Surgical Management of Concomitant Gallbladder and Common Bile Duct Stones

**DOI:** 10.1155/2015/165068

**Published:** 2015-09-01

**Authors:** J. H. Darrien, K. Connor, A. Janeczko, J. J. Casey, S. Paterson-Brown

**Affiliations:** Department of General & Upper GI Surgery, Royal Infirmary of Edinburgh, 51 Little France Crescent, Edinburgh EH16 4SA, UK

## Abstract

*Background*. The management of choledocholithiasis has evolved from open common bile duct exploration (OCBDE) to therapeutic endoscopic retrograde cholangiopancreatography (ERCP) to laparoscopic common bile duct exploration (LCBDE). Each entails a degree of difficulty. *Aim*. To review 5-year results of bile duct exploration in an UGI unit. *Methods*. Common bile duct explorations (CBDEs) performed between January 2008 and January 2013 were identified from a prospectively collected clinical audit system and results reviewed retrospectively. *Results*. 216 CBDEs were performed, 119 (55%) as an emergency and 52 (24%) following failed ERCP. Open CBDE (OCBDE) was performed primarily in 34/216 (16%) patients and attempted laparoscopically in 182 (84%). Fifty nine (32%) Laparoscopic CBDEs (LCBDEs) were converted to OCBDE. Of the remaining 123 LCBDEs, 51 (41%) primary choledochotomies and 72 (59%) primary transcystic CBDEs (TC-CBDEs) were performed. Forty nine (68%) TC-CBDEs were considered successful and 23 (32%) failed. Fifteen failed TC-CBDEs were converted to a successful laparoscopic choledochotomy. Ductal clearance was achieved in 187/216 (87%) patients and retained stones were identified in 20/123 (16%) LCBDEs. Complications occurred in 52/216 (24%) patients. There were 8/216 (4%) bile leaks requiring further intervention. Postoperative ERCP was carried out in 32/216 (15%) patients and 9/216 (4%) required relaparoscopy/laparotomy. No patient died. *Conclusions*. Successful management of choledocholithiasis requires a breadth of laparoscopic and endoscopic expertise.

## 1. Introduction

The management of concomitant gall bladder and common bile duct (CBD) stones has evolved significantly over the past 20 to 30 years. In the era of open surgery, open common bile duct exploration (OCBDE) would be performed if any common bile duct (CBD) stones were identified at cholangiography. Following the introduction and rapid uptake of endoscopic retrograde cholangiopancreatography (ERCP), OCBDE was reserved for patients who failed ERCP.

Following the introduction of laparoscopic cholecystectomy (LC) [1] there has been a gradual increase in Laparoscopic CBDE (LCBDE) which has been shown by a few enthusiasts to be as effective at CBD clearance and associated with reduced hospital stay compared to preoperative ERCP followed by LC [2–5]. However LCBDE, either using the transcystic route (TC-LCBDE) or via a choledochotomy, does involve more advanced laparoscopic skills, often including flexible choledochoscopy, and as a result the default procedure in many hospitals remains to be ERCP either before or after laparoscopic cholecystectomy. Such is the reliance upon ERCP that in some centres surgery for CBD stones is now considered a lost art [6]. Although ERCP alone may be appropriate for many elderly patients with significant comorbidity, it is likely that many more patients could benefit from the totally laparoscopic approach, including the elderly [7]. The aim of this study was to review the overall results of the surgical management of CBD stones, performed by a variety of upper GI surgeons in one hospital, in order to identify the success or otherwise of the various techniques employed.

## 2. Methods

All patients with concomitant gall bladder and CBD stones who underwent surgery in the Royal Infirmary of Edinburgh (RIE) between January 2008 and January 2013 were identified using the Lothian Surgical Audit (LSA) clinical audit system. Information for LSA is collected prospectively including patient demographics, diagnoses, operative interventions, operating surgeon, and date of discharge. Diagnosis and operative intervention are coded at the point of entry into LSA which allows users to identify specific patient cohorts, such as those who have undergone a surgical common bile duct exploration. The identification of this cohort from LSA allowed patient information inclusive of demographics, presentation, investigation, management, and primary and secondary outcome measures, that is, ductal clearance, complication rates, and inhospital mortality, to be reviewed retrospectively from electronic patient records. Longer term follow-up data was reviewed using the hospital electronic (TRAK) records to identify later problems. The decision on management of each individual patient was left to the treating surgeon.

## 3. Results

During the 5-year period between January 2008 and January 2013, 216 patients underwent surgical CBDE, of whom 152 (70%) were female. Patients' age ranged from 14 to 92 years. One hundred and nineteen of the 216 (55%) were performed as part of an emergency admission. Reasons for presentation were biliary colic 119 (54%), acute cholecystitis 32 (15%), acute pancreatitis 23 (11%), cholangitis 21 (10%), and jaundice 17 (8%). Three (1%) patients were found to have stones incidentally during workup for hepatectomy, liver transplantation, and head and neck malignancy. There were no data on the reasons for presentation for one patient. All but two patients (1%) had abnormal liver function tests (LFTs) and 184/216 (85%) patients had their CBD stones diagnosed radiologically preoperatively. In the remaining 32 patients CBD stones were detected by intraoperative cholangiography (IOC).

Fifty-two patients (24%) came to surgical treatment following a total of 66 failed ERCPs; 38 (73%) underwent a single failed ERCP, 13 (25%) underwent two failed ERCPs, and 1 patient (2%) underwent three failed ERCPS.

Primary OCBDE was performed in 34/216 (16%) patients. Primary LCBDE was attempted in 182 (84%) patients, with 59 (32%) converted to open surgery due to failure to extract the stone in 21 (36%) patients, difficult anatomy in 20 (34%), and adhesions in 18 (30%). As a result a total of 93/216 (43%) OCBDEs and 123 LCBDEs (57%) were performed.

Of the 123 LCBDEs, 51 (41%) primary choledochotomies were carried out and 72 (59%) primary transcystic CBDEs (TC-CBDEs) were attempted, of which 23 (32%) failed. Reasons for failure included an inability to negotiate the cystic duct in 15 (65%) patients and an inability to engage the stone in 6 (26%) patients and in 2 (9%) patients the stone was too large to be removed via the cystic duct. Fifteen of the failed TC-CBDEs were converted successfully to a laparoscopic choledochotomy bringing the total number of laparoscopic choledochotomies performed to 66. In eight of the failed transcystic explorations the stones were considered small enough to leave and be managed expectantly. Six ultimately required postoperative ERCP. Of the total 157 choledochotomies (open and laparoscopic) performed, 109 (69%) were closed primarily with sutures and 41 (26%) over a T-tube; 4 patients were converted to a hepatic-jejunostomy and 3 to a choledochoduodenostomy. These last 7 patients were all managed in conjunction with the local specialist hepatobiliary-pancreatic surgeons.

An overall ductal clearance rate of 87% (187/216) was achieved; 90% for OCBDES with 9/93 (10%) retained stones and 84% for LCBDES with 20/123 (16%) retained stones. Ductal clearance rates varied further within the LCBDE group. Ductal clearance rates of 85% followed a choledochotomy and 82% followed a TC-CBDE. The ductal clearance rate of 82% following TC-CBDE includes the 8 failed TC-CBDEs whereby an active decision was made not to progress to either choledochotomy or open surgery but rather to manage the stones expectantly.

Postoperative bile leaks requiring intervention occurred in 8 (4%) patients (4 in each of the LCBDE and OCBDE groups). Six were managed operatively as follows: (1) Bile leak after formation of hepaticojejunostomy at the primary operation was managed by revision of the hepaticojejunostomy. (2) High volume bile leak from choledochotomy site was managed by formation of hepaticojejunostomy. (3) Bile leak from choledochotomy from unrecognised pancreatic lesion was managed by double bypass. (4) Bile leak from choledochotomy was managed by relaparoscopy and insertion of T-tube. (5) Bile leak from choledochotomy was managed by relaparoscopy and suture repair. (6) Bile leak from choledochotomy was managed by relaparoscopy and drainage. Two bile leaks were managed by ERCP and sphincterotomy with insertion of pigtail stent.

Overall complications inclusive of retained stones occurred in 81/216 (38%) patients, 41/93 (44%) after OCBDE and 40/123 (32%) after LCBDE (see Table 1). If retained stones are excluded, the procedure related complications rates fall to 32/93 (34%) after OCBDE and 20/123 (16%) after LCBDE. Seven “other” complications noted in Table 1 include 2 urinary retentions, 1 clostridium difficile infection, 1 T-tube which fell out unexpectedly, 1 leak from a hepaticojejunostomy requiring revision, 1 bile leak requiring hepaticojejunostomy formation, and 1 unexplained systemic inflammatory response. To manage these complications 29/216 (13%) patients received further medical therapy, 32/216 (15%) patients underwent postoperative ERCP, 10 of which had already undergone preoperative ERCP, and 9/216 (4%) patients underwent laparoscopy or laparotomy. There were no deaths.

## 4. Discussion

The aim of this study was to review practice and performance in the surgical management of CBD stones at a single centre and within a subspecialist upper GI unit. Although results from single surgeons' series in the literature are excellent [2, 3] including in the emergency setting [8], this study describes results from a number of surgeons (all with a different level of experience) and included both elective and emergency patients. As such patients included in this cohort were not subject to standardisation by protocol, although in general choledochotomies were only performed where a minimum CBD diameter of 8 mm was identified. Decisions to perform primary OCBDE or LCBDE were made at the discretion of the individual operating surgeon, as was the use of additional intraoperative cholangiography in the 184/216 (85%) of patients who had their CBD stones diagnosed radiologically preoperatively. Operative decisions took into account both patient and surgeon factors with the objective of performing safe and successful surgery.

The results reported by two review articles [2, 3] have demonstrated that in experienced hands single-stage management of concomitant gall bladder and CBD stones in the form of LCBDE can achieve equivalent rates of ductal clearance, morbidity, and mortality to that of two-stage management in the form of laparoscopic cholecystectomy (LC) and either pre- or postoperative ERCP, with ductal clearance rates in the order of 84–97%, a conversion rate from LCBDE to either OCBDE or ERCP of 12%, a morbidity rate of 4–16%, and a mortality rate of 0–0.8%.

**Figure 1:**
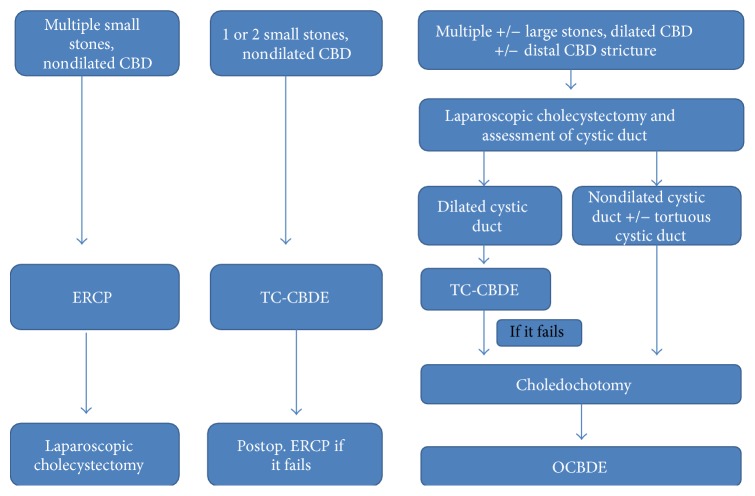
Suggested algorithm for the surgical management of choledocholithiasis.

We report equivalent rates of ductal clearance to that described in these other studies but variability in success within the LCBDE group. CBDE performed using a choledochotomy and choledochoscopy had a ductal clearance rate of 85% compared to 82% with transcystic CBDEs, the latter falling short of what is described in the literature. Furthermore, we noted that within the primary TC-CBDE group 32% of transcystic explorations fail due to an inability to negotiate the cystic duct requiring conversion to choledochotomy. These findings are particularly relevant as TC-CBDE is often perceived by surgeons less experienced in LCBDE as an easier option. They therefore need to be prepared to make the decision to either move on to laparoscopic choledochotomy and choledochoscopy or OCBDE or take no further action and arrange a postoperative ERCP. Preoperative investigation by means of MRCP can facilitate this operative decision making and in particular inform upon the need to proceed to choledochotomy. Radiological criteria for a choledochotomy are multifactorial and include information regarding the size of the stone, number of ductal stones, and presence of any distal CBD stricture. A patient with a dilated CBD (>8 mm) and with several large stones and a distal stricture is highly likely to fail ERCP and so LCBDE is more appropriate. A patient with a single small stone in a relatively nondilated CBD may be better managed by ERCP. Patients in between these two examples can be managed either way, but this will depend upon local expertise. Long tortuous cystic ducts with low insertions will likely make TC-CBDE more challenging if not impossible. Similarly multiple small stones in a nondilated CBD will be difficult and time consuming to remove using TC-CBDE with significant risk of displacing some of the stones into the proximal CBD which then cannot be retrieved. All these factors need to be taken into account when deciding the best approach. A suggested algorithm is shown in Figure 1.

Our study had a higher conversion rate (32%) of LCBDEs to open surgery as opposed to the 12% conversion rate described in the literature [2, 3]. The majority of these (64%) occurred due to adhesions or “challenging anatomy,” which might be related to the high number of procedures carried out on “emergency” admissions or following a failed preoperative ERCP [9], as well as the difference in experience of some of the surgeons. Furthermore as some of these patients had been referred from other hospitals, they may have been considered to be at a higher risk from the onset, thus explaining this observed difference. Improved patient pathways and reorganisation of resource may help direct patients straight to those surgeons with appropriate expertise and so facilitate more LCBDEs. In experienced hands a morbidity of 4–16% is described for LCBDEs [2, 3]. These studies do not include retained stones as a complication. If retained stones are excluded as a complication from our study, the morbidity of 20/123 (16%) is similar.

Ductal clearance is best confirmed after choledochotomy with choledochoscopy and after TC-CBDE with a completion cholangiogram. Despite these measures it is possible to “miss” stones although during the time of this study it is likely that most will have represented and have been included in our overall analysis. Due to the current electronic patient record system in use in our region, we were able to review the records of all patients in the series to confirm whether or not they had been readmitted with further problems secondary to retained stones in any of the 3 hospitals in our region. As such we believe our rate of retained stones to be reasonably accurate.

Postoperative bile leak after choledochotomy may be regarded as the most concerning of early complications. Like other units, T-tubes are rarely used in our unit [10], and a drain is left routinely adjacent to the choledochotomy site.

Although CBD strictures are often regarded as the most concerning of potential late complications, there were none in this study. As the regional and national hepatobiliary service is in our hospital it is very unlikely that any patient developed a stricture without our knowledge.

In our study 24% of patients proceeded to surgical common bile duct exploration following a number of failed ERCPs. These are considered to be complex cases [9] in which the success rate of LCBDE decreases, and the conversion to OCBDE is higher. This may have contributed to our 32% conversion rate to OCBDE. Although in our hospital the majority of patients requiring emergency admission with choledocholithiasis are admitted under the care of the surgical team, many “elective” patients with suspected choledocholithiasis are often initially referred to the medical gastroenterologists. This may account for differences in patient management, with patients admitted under the care of gastroenterology more likely to be managed endoscopically in the first instance, with those failing ERCP being referred for LCBDE. Whereas patients admitted first to the surgical team being more likely to go straight to surgery if appropriate. With increasing experience in LCBDE it is hoped that the patient pathway can be improved to avoid unnecessary ERCPs and direct those patients suitable for LCBDE straight to the surgical team. Unfortunately we do not have the data regarding those patients treated for CBD stones primarily by ERCP, only those who failed and were referred to surgery.

## 5. Conclusion

Successful management of choledocholithiasis requires a breadth of skills and expertise. Single-stage laparoscopic surgery can produce equivalent rates of ductal clearance to that obtained using a two-stage approach but requires surgical experience and optimisation of patient pathways to achieve the best results. While TC-CBDE is a useful technique with a relatively low morbidity, it does have a high failure rate and pre-operative imaging with MRCP is useful in identifying those patients in whom a choledochotomy may be required. This in turn may be helpful in choosing both the surgeon and the type of surgery.

## Figures and Tables

**Table 1:** Complications.

Complication	OCBDE (*n* = 93)	Clavien-Dindo Classification	Percentage (%)	LCBDE (*n* = 123)	Clavien-Dindo Classification	Percentage (%)
Retained stones	9	IIIa (7) IIIb (2)	10	20	I (2) IIIa (18)	16

Bile leak	4	IIIb (4)	4	4	IIIa (2) IIIb (2)	3

Respiratory	9	I (2) II (7)	10	4	I (3) II (1)	3

Cardiac	4	I (1) II (3)	4	0		0

Wound infection	7	II (6) IIIb (1)	8	1	II (1)	1

Acute pancreatitis	0		0	4	I (1) IIIa (3)	3

Jaundice	0		0	2	II (1) IIIa (1)	2

Collections	2	II (2)	2	4	I (2) II (1) IIIb (1)	3

Others	6	I (3) II (3)	6	1	IIIb (1)	1

Total	41		44	40		32
